# Concurrent dilution and amplification effects in an intraguild predation eco-epidemiological model

**DOI:** 10.1038/s41598-023-33345-2

**Published:** 2023-04-20

**Authors:** Enith A. Gómez-Hernández, Felipe N. Moreno-Gómez, Moisés Bravo-Gaete, Fernando Córdova-Lepe

**Affiliations:** 1 Doctorado en Modelamiento Matemático Aplicado, Universidad Católica del Maule, Talca, Chile; 2 Departamento de Biología y Química, Facultad de Ciencias Básicas, Universidad Católica del Maule, Talca, Chile; 3 Departamento de Matemática, Física y Estadística, Facultad de Ciencias Básicas, Universidad Católica del Maule, Talca, Chile

**Keywords:** Ecology, Systems biology, Diseases

## Abstract

The dilution and amplification effects are important concepts in the field of zoonotic diseases. While the dilution effect predicts that pathogen prevalence is negatively correlated with increased species diversity, the opposite trend is observed when the amplification effect occurs. Understanding how interspecific interactions such as predation and competition within a community influence disease transmission is highly relevant. We explore the conditions under which the dilution and amplification effects arise, using compartmental models that integrate ecological and epidemiological interactions. We formulate an intraguild predation model where each species is divided into two compartments: susceptible and infected individuals. We obtained that increasing predation increases the disease transmission potential of the predator and the density of infected individuals, but decreases the disease transmission potential of the prey, as well as their density. Also, we found that interspecific competition always helps to decrease the number of infected individuals in the population of the two species. Therefore, dilution and amplification effects can be observed simultaneously but depending on different types of cological interactions.

## Introduction

Most infectious diseases in recent decades, including the COVID-19 pandemic, have been caused by zoonotic pathogens^1^. Recent outbreaks of these diseases are related to anthropogenic activities that have impacted biodiversity^2^. New efforts aim to identify solutions that benefit human health and the environment, including understanding how wildlife alterations could determine the occurrence of zoonoses^3,4^. The connection between biodiversity and zoonotic disease emergence is contrasting. It is possible that maintaining biodiversity protects against disease through a dilution effect. However, an opposite trend is also possible through an amplification effect, resulting in an increased zoonotic risk^3,5^. These contradictory results arise from studying theoretical and empirical models that produce negative (dilution) and positive (amplification) relationships between pathogen prevalence and biodiversity^6,7^. Since these concepts are defined as a change in overall transmission, no clear framework explicitly shows the factors leading to a reduction or increase in disease. Transmission is difficult to quantify and apparently depends on multiple factors^8^. Ecological interactions between species are important factors in understanding dilution and amplification effects. In principle, interactions such as competition may promote dilution by reducing host abundance. However, in the case of predation, the expected outcome may depend on the trophic position of the reservoir. Thus, a mechanistic approach involving both interactions is needed for a better understanding of these effects^9,10^.

Predation and competition are the most studied interspecific interactions in ecology, partly because they constitute key factors involved in community structuring^11,12^. These interactions were mathematically enunciated in the 1920s by Lotka and Volterra. They independently proposed a model to describe the relationship between two species using the same resource, and then shifted their attention from competition to the effects of predation on population growth^13^. These models constitute a basis for integrating epidemiological processes into ecological systems, giving rise to eco-epidemiological models that describe the interspecific interaction between species as well as the dynamics of disease transmission^14^. Consequently, eco-epidemiology, as a new branch of mathematical biology, has become a theoretical tool for analyzing problems related to wildlife diseases^15^. Many researchers have studied the eco-epidemiological predator-prey model with diseases in the prey^16–20^, and with a disease in the predator^21–24^. The priority of works mentioned above has been to determine the interplay between infections and ecological dynamics.

Some authors have already approached the study of dilution and amplification effects through mathematical modeling^5,7,25^. In^7^, they use a susceptible-infected mathematical model and a unique long-term, high-resolution, multisite dataset to study the dynamics of infection in the deer mouse population, the host of Sin Nombre Virus (SNV). The authors fitted the model for each site. They observed that areas with higher diversity lead to the dilution effect due to decreased host density and, simultaneously, lead to the amplification effect by increasing transmission speed. In^25^, they formulated an eco-epidemiological model for the transmission of the SNV in the population of mice, which is a competition model where one of the species is infected, allowing to study how additional species can act as a dilution or amplification agents. The authors stress the importance of competing species to understand their relationship with disease prevalence better. In^5^, they propose an eco-epidemiological model where the relationship between two species is associated with resource competition and where each species is a host of the same infectious agent. They used the model to postulate dilution effects to understand the changes in dynamics, interpreting the decrease in biodiversity as a reduction in the density of one of the species.

The studies mentioned in the last paragraphs formulated eco-epidemiological models involving predation or competition. Here, we aim to integrate both interaction types by exploring an eco-epidemiological dynamic incorporating intraguild predation (IGP). IGP refers to a type of ecological interaction that occurs when two or more species that compete because they belong to the same guild (i.e., they occupy the same trophic level and use the same resources) also interact through direct predation. One common mathematical model used to study IGP is a modification of the Lotka–Volterra competition model which includes terms that account for the predation interactions between the species^26^. It is well known that IGP plays an important role in community structure, but how IGP affects the dynamics of a disease is an emerging research focus^14^. In our modeling approach, we assume distinct diseases that affect the prey and the predator, allowing us to obtain more specific conclusions associated to the position of the reservoir. Furthermore, we quantify the dilution and amplification effects by the density of infected prey and predator populations and by their basic reproductive numbers, which is a key measure for estimating the ability of the new pathogen to spread. Our analyses aim to improve the understanding of factors influencing zoonotic disease transmission.

The article is structured as follows: In Section “The model”, we derive the eco-epidemiological model, assuming an IGP interaction and different diseases for each species. In Section “Dynamics of the populations without disease”, we qualitatively analyze the IGP model without the disease. Then, in Section “Dynamics of the populations with disease”, we analyze the IGP model with disease and compared the results with the model studied in Section “Dynamics of the populations without disease”. Finally, in Section “Discussion”, we report the main conclusions of the study.

## The model

In this section, we begin with an IGP model to identify the specific mechanisms by which species diversity may decrease or increase disease risk. It is a model of competition between two populations $N_1$ and $N_2$, where in addition $N_1$ is a predator of $N_2$. The assumptions of the model are:

i. The predator $N_1$ has a generalist feeding strategy. Therefore, even in the absence of prey $N_2$, the predator population $N_1$ follows a logistic growth with carrying capacity $k_1$ and intrinsic growth rate $r_1$.
ii. In the absence of predators $N_1$, the prey population $N_2$ follows logistic growth with carrying capacity $k_2$ and intrinsic growth rate $r_2$.
iii. Species $N_1$ and $N_2$ compete for interference, *p* is the interspecific pressure exerted by the $N_2$ onto the $N_1$ and *q* is the interspecific pressure exerted by the $N_1$ onto the $N_2$.
iv. The type I Holling functional response is chosen to represent the per capita feeding rate of the predator on the prey. We denote by *a* the efficiency of predation and by $\varepsilon$ the conversion efficiency.

In view of the above assumptions, extending the notation of $N_1$ and $N_2$ for the respective abundances, the model takes the following form:

1 $$\begin{aligned} \begin{aligned} \frac{dN_1}{dt}&= r_1N_1\left( 1-\frac{N_1+pN_2}{k_1}\right) +\varepsilon aN_1N_2, \\ \frac{dN_2}{dt}&= r_2N_2\left( 1-\frac{N_2+qN_1}{k_2}\right) -aN_1N_2. \end{aligned} \end{aligned}$$

**Figure 1:**
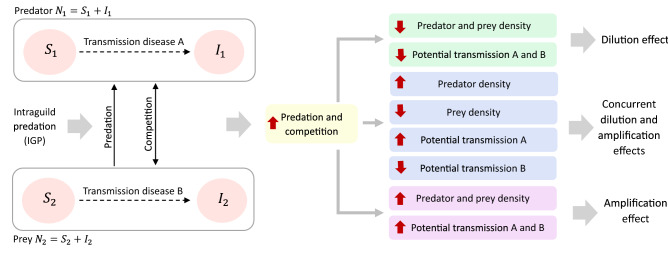
Schematic representation of an eco-epidemiological model and how competition and predation could affect the transmission and prevalence of a directly transmitted disease. Intraguild predation combines competition and predation between $N_1$ and $N_2$ species. Each species has a disease, the predators having disease A and the prey having disease B. The dotted arrow indicates disease transmission. The single-headed solid arrow indicates predation. The double-headed arrow represents interspecific competition. The direction of the arrowhead shows the flow of energy. Red upward arrows indicate an increase, and downward arrows indicate a decrease (adapted from Luis et al.^7^).

One way to assess the risk of zoonosis is to study the components of the probability of a pathogen jumping into humans. Obviously, people must have contact with the infected species. However, this aspect is outside the scope of this study, and we focus on other events necessary for a zoonosis to occur. Specifically, we will consider the potential for a disease transmission between individuals of a species and the size of the infected population of that species as a function of ecological parameters as shown in Fig. 1. For ease of exposition, we modeled pathogen spreads using a susceptible-infectious model, then divide $N_1$ and $N_2$ populations according to their epidemiological status, assuming that each species is a potential host of a different infectious agent. The model formulation assumes that the ecological interactions between $N_1$ and $N_2$ are not affected by the epidemiological status of individuals, i.e., infected individuals do not differ in their competitive strength and predation efficiency. The eco-epidemiological model follows the dynamics of the system (1), in which the population $N_{i}$ is compartmentalized, with respect to the disease into susceptible $S_i$ and infective $I_i$ states, so that $N_i=S_i+I_i$ (for $i \in \{1,2\}$). The model is given by:

2 $$\begin{aligned} \left\{ \begin{array}{cccccc} \tiny \textbf{Variation}&{} &{} \tiny { \textbf{Intrinsic Growth}} &{} \tiny { \textbf{Intra{-}inter Competition}}&{} \tiny \textbf{Predation} &{} \tiny \textbf{Contagion}\\ dS_1/dt &{}=&{} b_1N_1-d_1S_1 &{}-(r_1/k_1)S_1(N_1+pN_2)&{}+\varepsilon aN_1 N_2&{}-\beta _1 S_1I_1\\ dI_1/dt &{}=&{} -d_1I_1&{}-(r_1/k_1)I_1(N_1+pN_2)&{} &{}+ \beta _1 S_1I_1\\ dS_2/dt &{}=&{} b_2N_2-d_2S_2&{}-(r_2/k_2)S_2(N_2+qN_1)&{}-aS_2 N_1&{}-\beta _2 S_2I_2 \\ dI_2/dt &{}=&{} -d_2I_2&{}-(r_2/k_2)I_2(N_2+qN_1)&{}-a I_2 N_1 &{}+\beta _2 S_2I_2. \end{array} \right. \end{aligned}$$

The parameters of the model of Eq. (2), are defined in the same way as the model Eq. (1). We know that the intrinsic growth rates are defined as $r_i=b_i-d_i$, where $b_i$ is the per capita birth rate and $d_i$ is the per capita mortality rate. In model of Eq. (2) it is necessary to show them explicitly so as not to alter the dynamics of $N_1$ and $N_2$.

## Results

### Dynamics of the populations without disease

In this section, we summarize the main results of the steady state and stability analysis of the IGP model without disease of Eq. (1). Table 1 shows the equilibria of the model, the existence conditions of each equilibrium, and the local asymptotic stability conditions. The theorem and proof that led to these results can be found as Supplementary analysis of the disease-free model online. In the results, we find the trivial equilibrium $E_0(0,0)$. As $E_0$ is unstable, it is meaningless in our case. We distinguish the following nontrivial cases.

#### Predator-only case

The equilibrium where the prey goes extinct while the predator reaches their carrying capacity is $E_1(k_1,0)$. Here we note that this case is always feasible. It is locally asymptotically stable when the following condition is satisfied. The reproductive potential of prey $N_2$ is less than the damage exerted by species $N_1$ through competition and predation when it reaches its carrying capacity, and is given by $r_2<\alpha _2k_1$ where $\alpha _2=r_2q/k_2+a$. From the above expression ($\alpha _2$), the first term is the competition parameter and the second term is the predation efficiency.

#### Prey-only case

The equilibrium where the predator goes extinct while the prey reaches its carrying capacity is $E_2(0,k_2)$. It is always feasible. It is locally asymptotically stable if the reproductive potential of $N_1$ is less than that of the benefit from predation and damage from the competition, i.e., $r_1<\alpha _1 k_2$, where $\alpha _1=r_1p/k_1-\varepsilon a$. We note that if $r_1p/k_1=\varepsilon a$, then there is a trade-off between competition and predation.

#### Coexistence case

The equilibrium where the two populations coexist is $E^*\left( N_1^*, N_2^*\right)$. It is a feasible equilibrium when the following two conditions are satisfied. The first condition describes that the negative effects the prey $N_2$ on the predator $N_1$ due to competition and predation benefit is not greater than the reproductive potential of $N_1$, this is $\alpha _1K_2<r_1$. The second condition describes that the negative effects resulting both from predation and competition of species $N_1$ on $N_2$ is not greater than the reproductive potential of $N_2$, this is $\alpha _2k_1<r_2$.

**Table 1:** Equilibria and their stability of model (1), where $\alpha _1=r_1p/k_1-\varepsilon a$ and $\alpha _2=r_2q/k_2+a$ (see Supplementary analysis of the disease-free model).

Equilibrium	Existence	Stability condition
$E_0(0,0)$	Always	Always unstable
$E_1\left( k_1,0\right)$	Always	Locally asymptotically stable if $r_2<\alpha _2 k_1$
$E_2(0,k_2)$	Always	Locally asymptotically stable if $r_1<\alpha _1 k_2$
$E^*\left( N_1^*, N_2^*\right)$	$\alpha _1k_2<r_1$, $\alpha _2k_1<r_2$ and $\alpha _1\alpha _2<\left( \frac{r_1}{k_1}\right) \left( \frac{r_2}{k_2}\right)$	Locally asymptotically stable if it exists

### Dynamics of the populations with disease

In this section, we summarize the main results of the steady state and stability analysis of the IGP model with disease of Equation (2). Table 2 shows the equilibria of the model, the existence conditions of each equilibrium, and the local asymptotic stability conditions. The theorem and proof that led to these results can be found as Supplementary analysis of the model with disease online. Taking into account that in the assumptions of the model (2), the disease does not affect any of the populations, when we add the susceptible and infected populations, the model (1) is naturally recovered. We have that the different possibilities of disease establishment only increases the equilibria concerning the IGP model without the disease. We note that equilibria $E_1$, $E_2$, $E_3$ and $E_4$ correspond to competitive exclusion equilibria, where either species reaches its own carrying capacity. To the stability conditions of the model (1), we add disease-related conditions. For this, we calculated the basic reproductive number of each disease using the spectral radius of the next-generation matrix^27^. The procedure can be found as Supplementary basic reproductive number online. In particular, we assume that $E_1$ is the infection-free equilibrium and $E_3$ is the endemic equilibrium when the predator reaches its carrying capacity. We calculate their respective basic reproductive number which is given by $R_0^{(1,1)}=\frac{\beta _1k_1}{b_1}$. In the same way, we proceed when the prey wins and calculate its basic reproductive number $R_0^{(1,2)}=\frac{\beta _2k_2}{b_2}$. In addition to the conditions in Table 2, the local asymptotic stability depends on the basic reproductive numbers.

**Table 2:** Equilibria and their stability of model (2), where $r_1=b_1-d_1$, $r_2=b_2-d_2$, $\alpha _1=r_1p/k_1-\varepsilon a$, $\alpha _2=r_2q/k_2+a$, $S_1^*=\frac{1}{\beta _1}+\frac{\varepsilon aN_2^*}{\beta _1}$ and $I_1^*=N_1^*-\frac{1}{\beta _1}-\frac{\varepsilon aN_2^*}{\beta _1}$ (see Supplementary analysis of the model with disease).

Equilibrium	Existence	Stability condition
$E_0\left( 0,0,0,0\right)$	Always	Always unstable
$E_1\left( k_1,0,0,0\right)$	Always	$r_2<\alpha _2k_1$ and $R_0^{(1,1)}<1$
$E_2\left( 0,0,k_2,0\right)$	Always	$r_1<\alpha _1k_2$ and $R_0^{(1,2)}<1$
$E_3\left( \frac{b_1}{\beta _1},k_1-\frac{b_1}{\beta _1},0,0\right)$	$R_{0}^{(1,1)}>1$	$r_2<\alpha _2k_1$ and $R_0^{(1,1)}>1$
$E_4\left( 0,0,\frac{b_2}{\beta _2},k_2-\frac{b_2}{\beta _2}\right)$	$R_{0}^{(1,2)}>1$	$r_1<\alpha _1k_2$ and $R_0^{(1,2)}>1$
$E_5\left( N_1^*,0, N_2^*,0\right)$	$\alpha _1k_2<r_1$, $\alpha _2k_1<r_2$	$R_{0}^{(2,1)}<1$ and $R_{0}^{(2,2)}<1$
	and $\alpha _1\alpha _2<\left( \frac{r_1}{k_1}\right) \left( \frac{r_2}{k_2}\right)$	
$E_6\left( S_1^*, I_1^*, N_2^*,0\right)$	$R_0^{(2,1)}>1$	$R_{0}^{(2,1)}>1$ and $R_{0}^{(2,2)}<1$
$E_7\left( N_1^*,0,\frac{b_2}{\beta _2},N_2^*-\frac{b_2}{\beta _2}\right)$	$R_0^{(2,2)}>1$	$R_{0}^{(2,1)}<1$ and $R_{0}^{(2,2)}>1$
$E^*\left( S_1^*, I_1^*, \frac{b_2}{\beta _2},N_2^*-\frac{b_2}{\beta _2}\right)$	$R_0^{(2,1)}>1$ and $R_0^{(2,2)}>1$	$R_{0}^{(2,1)}>1$ and $R_{0}^{(2,2)}>1$

**Figure 2:**
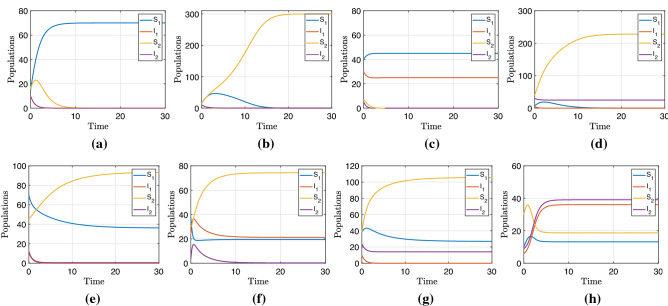
Numerical example of each of the equilibria. (**a**) Equilibrium $E_1$ with $R_0^{(1,1)}=0.77$, (**b**) Equilibrium $E_2$ with $R_0^{(1,2)}=0.30$, (**c**) Equilibrium $E_3$ with $R_0^{(1,1)}=1.55$, (**d**) Equilibrium $E_4$ with $R_0^{(1,2)}=3.00$, (**e**) Equilibrium $E_5$ with $R_0^{(2,1)}=0.13$ and $R_0^{(2,2)}=0.10$, (**f**) Equilibrium $E_6$ with $R_0^{(2,1)}=2.13$ and $R_0^{(2,2)}=0.06$, (**g**) Equilibrium $E_7$ with $R_0^{(2,1)}=0.02$ and $R_0^{(2,2)}=4.60$, (**h**) Equilibrium $E^*$ with $R_0^{(2,1)}=2.66$ and $R_0^{(2,2)}=5.11$. Simulations are conducted using MATLAB *ode45*. Numerical values for the simulations can be found as Supplementary numerical values.

We performed numerical simulations that exemplify each of the equilibria from Table 2. The software used in the simulations was Matlab, with the routine ode45. This solver is based on an explicit Runge–Kutta (4,5) formula, the Dormand-Prince pair. That means the numerical solver ode45 combines fourth and fifth order methods. These vary the step size, choosing it at each step an attempt to achieve the desired accuracy. Our model has nine equilibria, one of which is the trivial one for which we have not done a simulation. The other equilibria correspond to each of the subfigures in Fig. 2. The first row of Fig. 2 shows simulations corresponding to each equilibrium where competitive exclusion exists. The equilibria $E_5$, $E_6$, $E_7$, and $E^*$ correspond to the coexistence of predators and prey. Evidently, $E_5$ is the infection-free equilibrium with coexistence, so before finding the stability conditions, we calculate the basic reproductive number of each disease.

3 $$\begin{aligned}{} & {} R_0^{(2,1)} = \frac{k_1\beta _1N_1^*}{d_1k_1+pr_1N_2^*+r_1N_1^*}, \end{aligned}$$

4 $$\begin{aligned}{} & {} R_0^{(2,2)} = R_0^{(1,2)} \left( \frac{N_2^*}{k_2}\right) =\left( \frac{\beta _2k_2}{b_2}\right) \frac{N_2^*}{k_2}. \end{aligned}$$

The stability condition of $E_5$ is that both basic reproductive numbers are less than 1. In the case of equilibrium $E_6$, we have that disease in predators is established while disease in prey is not established, so the stability condition is $R_0^{(2,1)}>1$ and $R_0^{(2,2)}<1$. In equilibrium $E_7$, the disease is only endemic in the prey population, and so here $R_0^{(2,1)}<1$ and $R_0^{(2,2)}>1$. The equilibrium $E^*$ where both predator and prey disease basic reproductive numbers must be greater than 1. Notably, the susceptible population prey in the equilibrium depends only on natality and the rate of disease transmission. In contrast, those infected depend on competition and predation parameters. The second row of Fig. 2 shows the simulations for the coexistence equilibria.

**Figure 3:**
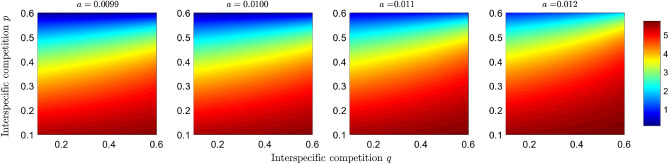
Effect of competition and predation on the basic reproductive number of the disease in the predators. Simulations plot the $R_0^{(2,1)}$ given in Eq. (3) as a function of the competition parameters *p* and *q* under different scenarios of predation efficiency *a*, where $(q,p)\in [0.1,0.6]\times [0.1,0.6]$. The color bar shows the numerical value of the basic reproductive number.

**Figure 4:**
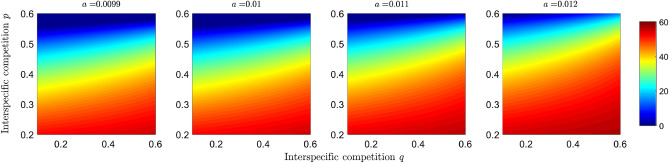
Simulation of the infected population of the predator at equilibrium as a function of the competition parameters *p* and *q* under different scenarios of predation efficiency *a*, where $(q,p)\in [0.1,0.6]\times [0.1,0.6]$. The colored bar represents the number of infected individuals.

To assess how competition and predation can give rise to the dilution or amplification effect, we explore the case of coexistence of all compartments of the model (2) and determine how the basic reproductive numbers change as a function of competition and predation parameters. We use the parameter values from the simulation shown in Fig. 2h. The Fig. 3 shows how the basic reproductive number of the predator disease $R_0^{(2,1)}$ changes as a function of the interspecific competition parameters *p* and *q*. We observe that the higher the interspecific competition exerted by the prey, the basic reproductive number decreases, and therefore the potential for transmission decreases. In Fig. 4, we observe a similar trend for the population of the infected predator $I_1$, and it is clearly observed how the competition exerted by $N_2$ decreases the number of infected predators at equilibrium. Now, when predation efficiency *a* increases, the region where the basic reproductive number is greater than 1 increases (see Fig. 3), and the number of infected increases (see Fig. 4).

**Figure 5:**
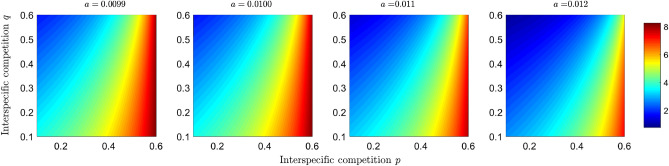
Effect of competition and predation on the basic reproductive number of the disease in the preys. Simulations plot the $R_0^{(2,2)}$ given in Eq. (4) as a function of the competition parameters *p* and *q* under different scenarios of predation efficiency *a*, where $(q,p)\in [0.1,0.6]\times [0.1,0.6]$. The color bar shows the numerical value of the basic reproductive number.

**Figure 6:**
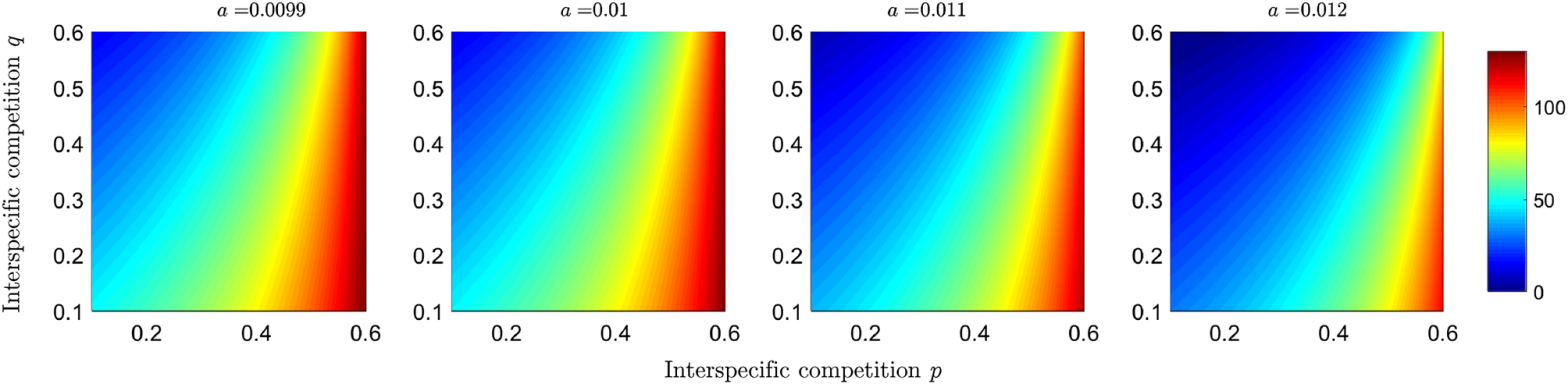
Simulation of the infected subpopulation of the prey at equilibrium as a function of the competition parameters *p* and *q* under different scenarios of predation efficiency *a*, where $(q,p)\in [0.1,0.6]\times [0.1,0.6]$. The colored bar represents the number of infected individuals.

In Figs. 5 and 6, we show the changes in the basic reproductive number of prey $R_0^{(2,2)}$ and the population of infected preys $I_2$ as a function of the interspecific competition parameters *p* and *q*, as well as some scenarios for different values of the predation efficiency *a*. It is observed that when predator interspecific competition increases, the basic reproductive number of the disease in prey decreases, as does the number of infected individuals. When predation increases, the region where $R_0^{(2,2)}>1$ decreases (see Fig. 5), and the number of individuals in equilibrium decreases (see Fig. 6).

In short, the objective of plotting the basic reproductive numbers of each disease (Figs. 3 and 5) as a function of competition and predation parameters is to determine the effect of interspecific interactions on disease transmission potential. In addition to taking into account the potential for disease transmission, we took into account the density of infected populations (Figs. 4 and 6) to study the effects of dilution and amplification.

## Discussion

Zoonotic diseases account for approximately 60% of all human infectious diseases, making them a severe public health problem^28^. This is why several researchers have suggested the importance of the One Health approach to address these diseases^29–32^. The approach encourages the integrated study of human, animal, and environmental health^33^. Therefore, analyzing reservoir dynamics in a community context is a fundamental part of the search for mechanisms to prevent pathogen transmission^34^. Indeed, the decrease in biodiversity modifies the trophic relationships that regulate populations, which may imply a change in the reservoirs and, therefore, a change in the dynamics of the disease^35^. Theoretical studies show that increasing host biodiversity reduces disease (dilution) or increases it (amplification) depending on interspecific interactions^10,36,37^. Then, a mechanistic approach that considers interspecific interactions is appropriate because of the wide variety of patterns it can predict^38^.

In this paper, we have proposed and analyzed an IGP model, assuming each species has a susceptible-infected disease type. Typical models in epidemiology generally consider closed populations; in a susceptible-infected model, this leads to all individuals becoming infected over time^39,40^. Here, we assume a model with vital dynamics where all newborns are susceptible and incorporate unequal birth and death rates. On the other hand, it should be noted that eco-epidemiological and ecological models with more than two species can be equivalent under certain conditions. In^41^, the authors show that a predator-prey model with infection in the prey can be studied as a model of exploitative competition and that disease in the predator leads to a tri-trophic food chain. Our eco-epidemiological model can lead to a model of two predator species and two prey species; the assumptions under which this analogy can be established are that infection does not alter predation preferences and that resources are energetically equivalent for the consumer. This equivalence is beneficial for the transfer of knowledge about the systems. Therefore, the conclusions of the model presented in this article can also contribute to the study of a model with two predators and two prey.

First, we analyzed the IGP model without the disease. This model has four equilibria for which we obtained sufficient ecological conditions on the parameters for which the system is locally and asymptotically stable around each equilibrium. The key parameters in this model are predation efficiency and interspecific competition. We then analyze the IGP model with disease, which has nine equilibria, and where we discover the key requirements on the parameters of the proposed system for both existence and local asymptotic stability. The results were given in terms of the ecological conditions found in the IGP model without disease and the basic reproductive number of each of the diseases. We obtained the typical threshold behavior in epidemiology: when the basic reproductive number is less than one, the disease disappears from the population, and when it is greater than one, the disease persists in time. The previous results allowed us to evaluate how competition and predation can produce the dilution and amplification effect. We obtained that increasing predation increases the disease transmission potential of the predator and the density of infected individuals, but decreases the disease transmission potential of the prey, as well as their density. So increased predation decreases the infection of one of the species, but increases them in the other. In addition, we found that interspecific competition always helps to decrease the number of infected individuals in the population of the two species. Our approach, that implements an IGP model, allowed us to assess simultaneously two crucial ecological interactions allowing to obtain deeper insight into disease dynamics.

Our approach differs from other works with eco-epidemiological models, in the sense that we proposed a model to study the effect of predation and competition on the dynamics of a disease. In general, previous works have focused on how the disease changes the dynamics of interactions in a community. In our case, our work aims to understand how competition and predation are related to the effects of dilution and amplification through two indicators: (i) the impact on basic reproductive numbers and (ii) the density of infected populations. Additionally, our interest was associated with reservoirs of zoonotic diseases unaffected by their epidemiological state. For example, hantavirus reservoirs, rodents without symptoms or changes in its dynamics by the virus^42^.

As further works, our model can be expanded to cover the dynamics of any wildlife disease, not just reservoirs. In particular, we can assume differentiated mortality between susceptible and infected individuals for those situations where disease infection decreases the capacity for predation and competition^43^. In conclusion, we have presented a model with two species in the simplest eco-epidemiological environment, allowing us to relate interspecific interactions with wildlife diseases. Together with the above, our method can be generalized to any number of species, and more complicated ecological and epidemiological dynamics, channeling to a more direct study of the effects of dilution and amplification with the species diversity.

## Supplementary Information

Supplementary Information.

## Data Availability

All data generated or analysed during this study are included in this published article and its Supplementary information files.
