# Supplementary material for: Concurrent dilution and amplification effects in an intraguild predation eco-epidemiological model
Source: Sci Rep. 2023 Apr 20;13:6425. doi: 10.1038/s41598-023-33345-2 (PMC10119278; doi:10.1038/s41598-023-33345-2)
Supplement: Supplementary file 1 — Supplementary Information. [file 41598_2023_33345_MOESM1_ESM.pdf]

Supplementary information

Concurrent dilution and amplification effects in an intraguild predation eco-epidemiological model

Supplementary analysis of the disease-free model

In this supplementary, we determine the equilibria of IGP model without disease and analyze its local stability.

Theorem 1.

(i) The trivial equilibrium $E_0(0,0)$ always exists and it is a always unstable.

(ii) The predator-only equilibrium $E_1(k_1,0)$ always exists and it is locally asymptotically stable if $r_2 < \alpha_2 k_1$, where $\alpha_2 = r_2 q/k_2 + a$.

(iii) The predator-only equilibrium $E_2(0,k_2)$ always exists and it is locally asymptotically stable if $r_1 < \alpha_1 k_2$, where $\alpha_1 = r_1 p/k_1 - \varepsilon a$.

(iv) The coexistence equilibrium $E^*(N_1^*, N_2^*)$ exists if $\alpha_1 k_2 < r_1$ and $\alpha_2 k_1 < r_1$. E^* is locally asymptotically stable if it exists.

Proof. To obtain the equilibria, we solve the nonlinear system obtained from equating the right-hand side of the model to zero. To show the asymptotic stability of the equilibria, we use the method of first approximation. The Jacobian matrix at a point $E(N_1,N_2)$ is

$$J(E) = \begin{bmatrix} r_1\left(1-\dfrac{2N_1}{k_1}\right) - \alpha_1 N_2 & -\alpha N_1 \\ -\alpha_2 N_2 & r_2\left(1-\dfrac{2N_2}{k_2}\right) - \alpha_2 N_1 \end{bmatrix}.$$

(i) The Jacobian matrix at $E_0(0,0)$ has two eigenvalues $\lambda_{01} = r_1$ and $\lambda_{02} = r_2$. Thus, the trivial equilibrium E_0 is unstable.

(ii) The Jacobian matrix at $E_1(k_1,0)$ has two eigenvalues $\lambda_{11} = -r_1$ and $\lambda_{12} = -\alpha_2 k_1 + r_2$, i.e., the boundary equilibrium E_1 is locally asymptotically stable if $r_2 < \alpha_1 k_1$.

(iii) The Jacobian matrix at $E_2(0,k_2)$ has two eigenvalues $\lambda_{21} = -\alpha_1 k_2 + r_1$ and $\lambda_{22} = -r_2$, i.e., the boundary equilibrium E_2 is locally asymptotically stable if $r_1 < \alpha_1 k_2$.

(iv) The coexistence equilibrium is $E^*(N_1^*, N_2^*)$ with

$$N_1^* = \frac{k_1 r_2 (r_1 - \alpha_1 k_2)}{r_1 r_2 - \alpha_1 \alpha_2 k_1 k_2}, \quad \text{and} \quad N_2^* = \frac{k_2 r_1 (r_2 - \alpha_2 k_1)}{r_1 r_2 - \alpha_1 \alpha_2 k_1 k_2},$$

which is positive if $\alpha_1 k_2 < r_1$, $\alpha_2 k_1 < r_2$ and $\alpha_1 \alpha_2 < \left(\frac{r_1}{k_1}\right)\left(\frac{r_2}{k_2}\right)$. Evaluating the Jacobian matrix in this equilibrium we have that $J(E^*)$ has the following characteristic equation

$$p(\lambda_*) = \lambda_*^2 + a_1 \lambda + a_0,$$

with

$$a_0 = \frac{r_1 r_2 (r_2 - \alpha_2 k_1)(r_1 - \alpha_1 k_2)}{r_1 r_2 - \alpha_1 \alpha_2 k_1 k_2}, \quad \text{and} \quad a_1 = \frac{r_1 r_2 [(r_1 - \alpha_1 k_2) + (r_2 - \alpha_2 k_1)]}{r_1 r_2 - \alpha_1 \alpha_2 k_1 k_2}.$$

The existence conditions of equilibrium E^* ensure that the coefficients a_0 and a_1 are positive. Then by the Routh-Hurwitz criterion the equilibrium E^* is locally asymptotically stable.

□

Supplementary analysis of the model with disease

In this supplementary, we determine the equilibria of the IGP model with disease in prey and predator and analyze its local stability.

Theorem 2.

(i) The trivial equilibrium $E_0(0,0,0,0)$ always exists and it is a always unstable.

(ii) The predator-only equilibrium $E_1(k_1,0,0,0)$ always exists and it is locally asymptotically stable if $r_2 < \alpha_2 k_1$ and $R_0^{(1,1)} < 1$, where $\alpha_2 = r_2 q/k_2 + a$ and $R_0^{(1,1)} = \beta_1 k_1/b_1$.

(iii) The predator-only equilibrium $E_2(0,0,k_2,0)$ always exists and it is locally asymptotically stable if $r_1 < \alpha_1 k_2$ and $R_0^{(1,2)} < 1$, where $\alpha_1 = r_1 p/k_1 - \varepsilon a$ and $R_0^{(1,2)} = \beta_2 k_2/b_2$.

(iv) The predator-only equilibrium $E_3(b_1/\beta_1, k_1 - b_1/\beta_1, 0, 0)$ exists if $R_0^{(1,1)} > 1$, and it is locally asymptotically stable if it exists and if $r_2 < \alpha_2 k_1$.

(v) The prey-only equilibrium $E_4(0,0,b_2/\beta_2, k_2 - b_2/\beta_2)$ exists if $R_0^{(1,2)} > 1$, and it is locally asymptotically stable if it exists and if $r_1 < \alpha_1 k_2$.

(vi) The coexistence equilibrium $E_5(N_1^*, 0, N_2^*, 0)$ exists if $\alpha_1 k_2 < r_1$ and $\alpha_2 k_1 < r_1$ and it is locally asymptotically stable if $R_0^{(2,1)} < 1$ and $R_0^{(2,2)} < 1$, where

$$R_0^{(2,1)} = \frac{k_1 \beta_1 N_1^*}{d_1 k_1 + p r_1 N_2^* + r_1 N_1^*},$$

$$R_0^{(2,2)} = R_0^{(1,2)} \frac{N_2^*}{k_2}.$$

(vii) The coexistence equilibrium $E_6(S_1^*, I_1^*, N_2^*, 0)$ exists if $R_0^{(2,1)} > 1$, and it is locally asymptotically stable if $R_0^{(2,1)} > 1$ and $R_0^{(2,2)} < 1$, here $S_1^* = \frac{1}{\beta_1} + \frac{\varepsilon a N_2^*}{\beta_1}$ and $I_1^* = N_1^* - \frac{1}{\beta_1} - \frac{\varepsilon a N_2^*}{\beta_1}$.

(viii) The coexistence equilibrium $E_7(N_1^*, 0, b_2/\beta_2, N_2^* - b_2/\beta_2)$ exists if $R_0^{(2,2)} > 1$, and it is locally asymptotically stable if $R_0^{(2,1)} < 1$ and $R_0^{(2,2)} > 1$.

(ix) The coexistence equilibrium $E^*(S_1^*, I_1^*, b_2/\beta_2, N_2^* - b_2/\beta_2)$ exists if $R_0^{(2,2)} > 1$ and $R_0^{(2,2)} > 1$, and it is locally asymptotically stable if it exists. Where $S_1^* = \frac{1}{\beta_1} + \frac{\varepsilon a N_2^*}{\beta_1}$ and $I_1^* = N_1^* - \frac{1}{\beta_1} - \frac{\varepsilon a N_2^*}{\beta_1}$.

Proof. We proceed in the same way as in the proof of Theorem 1. The Jacobian matrix at a point $E(S_1, I_1, S_2, I_2)$ is

$$\bar{J}(E) = \begin{bmatrix} J_{11} & b_1 - \frac{r_1 S_1}{k_1} + \varepsilon a N_2 - \beta_1 S_1 & -\frac{r_1 p S_1}{k_1} + \varepsilon a N_1 & -\frac{r_1 p S_1}{k_1} + \varepsilon a N_1 \\ \beta_1 I_1 - \frac{r_1 I_1}{k_1} & J_{22} & -\frac{r_1 p I_1}{k_1} & -\frac{r_1 p I_1}{k_1} \\ -\alpha_2 S_2 & -\alpha_2 S_2 & J_{33} & b_2 - \frac{r_2 S_2}{k_2} - \beta_2 S_2 \\ -\alpha_2 I_2 & -\alpha_2 I_2 & \beta_2 I_2 - \frac{r_2 I_2}{k_2} & J_{44} \end{bmatrix}.$$

where

$$J_{11} = r_1 - \frac{r_1 N_1}{k_1} - \frac{r_1 S_1}{k_1} - \alpha_1 N_2 - \beta_1 I_1,$$
$$J_{22} = \beta_1 S_1 - d_1 - \frac{r_1 N_1}{k_1} - \frac{r_1 p N_2}{k_1} - \frac{r_1 I_1}{k_1},$$
$$J_{33} = r_2 - \frac{r_2 N_2}{k_2} - \frac{r_2 S_2}{k_2} - \alpha_2 N_1 - \beta_2 I_2 \text{ and}$$
$$J_{44} = \beta_2 S_2 - d_2 - \frac{r_2 N_2}{k_2} - \frac{r_2 I_2}{k_2} - \alpha_2 N_1.$$

(i) The Jacobian matrix at $E_0(0,0,0,0)$ has four eigenvalues $\lambda_{01} = r_1$, $\lambda_{02} = -d_1$, $\lambda_{03} = r_2$ and $\lambda_{04} = -d_2$. Thus, the trivial equilibrium E_0 is unstable.

(ii) The Jacobian matrix at $E_1(k_1,0,0,0)$ has four eigenvalues $\lambda_{11} = -r_1$, $\lambda_{12} = \beta_1 k_1 - d_1 - r_1$, $\lambda_{13} = -\alpha_2 k_1 + r_2$ and $\lambda_{14} = -\alpha_2 k_1 - d_2$ i.e., the boundary equilibrium E_1 is locally asymptotically stable if $r_2 < \alpha_1 k_1$ and $\beta_1 k_1 < d_1 + r_1$. Since E_1 is an infection-free equilibrium one can rewrite the second stability condition as $R_0^{(1,1)} < 1$, where $R_0^{(1,1)}$ represents the basic reproductive number of predators in the absence of prey.

(iii) The Jacobian matrix at $E_2(0,0,k_2,0)$ has four eigenvalues $\lambda_{21} = -\alpha_1 k_2 + r_1$, $\lambda_{22} = -(pk_2 r_1 + d_1 k_1)/k_1$, $\lambda_{23} = -r_2$ and $\lambda_{24} = \beta_2 k_2 - d_2 - r_2$ i.e., the boundary equilibrium E_2 is locally asymptotically stable if $r_1 < \alpha_1 k_2$ and $\beta_2 k_2 < d_2 + r_2$. Similar to item (ii) we can rewrite the second condition as $R_0^{(1,2)} < 1$, here $R_0^{(1,2)}$ represents the basic reproductive number of prey in the absence of predators.

(iv) By straightforward calculation we obtain that the eigenvalues of the matrix $\bar{J}(E_3)$ are: $\lambda_{31} = r_2 - \alpha_2 k_1$, $\lambda_{32} = -d_2 - \alpha_2 k_1$, $\lambda_{23} = -r_1$ and $\lambda_{34} = b_1 - \beta_1 k_1$. It follows from the definition $R_0^{(1,1)}$ that E_4 is locally asymptotically stable if $r_2 < \alpha_2 k_1$ and $R_0^{(1,1)} > 1$.

(v) The eigenvalues of $\bar{J}(E_4)$ are $\lambda_{41} = r_1 - \alpha_1 k_2$, $\lambda_{42} = -(pk_2 r_1 + d_1 k_1)/k_1$, $\lambda_{43} = -r_2$ and $\lambda_{44} = b_2 - \beta_2 k_2$. Therefore, the equilibrium E_5 is locally asymptotically stable if $r_1 < \alpha_1 k_2$ and $R_0^{(1,2)} > 1$.

(vi) The eigenvalues of $\bar{J}(E_5)$ are

$$\lambda_{51} = \left(R_0^{(2,1)} - 1\right)\left(d_1 + \frac{r_1 p N_2^*}{k_1} + \frac{r_1 N_1^*}{k_1}\right),$$
$$\lambda_{52} = b_2\left(R_0^{(2,2)} - 1\right) \text{ and}$$
$$\lambda_{53,54} = -\frac{1}{2}\frac{1}{r_1 r_2 - \alpha_1 \alpha_2 k_1 k_2}\left(b^2 \pm b\sqrt{1 - \frac{4c}{b^2}}\right),$$

where,

$$b = r_1 r_2(r_1 - \alpha_1 k_2) + r_1 r_2(r_2 - \alpha_2 k_1) \text{ and}$$
$$c = r_1 r_2(r_1 r_2 - \alpha_1 \alpha_2 k_1 k_2)(r_1 - \alpha_1 k_2)(r_2 - \alpha_2 k_1).$$

If $b^2 > 4c$ then both eigenvalues are negative and if $b^2 < 4c$ then the real part of the eigenvalues is negative. Therefore, the equilibrium E_6 is locally asymptotically stable if $R_0^{(2,1)} < 1$ and $R_0^{(2,2)} < 1$.

(vii) The eigenvalues of $\bar{J}(E_6)$ are

$$\lambda_{61} = \left(1 - R_0^{(2,1)}\right)\left(d_1 + \frac{r_1 p N_2^*}{k_1} + \frac{r_1 N_1^*}{k_1}\right),$$
$$\lambda_{62} = b_2\left(R_0^{(2,2)} - 1\right) \text{ and}$$
$$\lambda_{63,64} = -\frac{1}{2}\frac{1}{r_1 r_2 - \alpha_1 \alpha_2 k_1 k_2}\left(b^2 \pm b\sqrt{1 - \frac{4c}{b^2}}\right),$$

where,

$$b = r_1 r_2(r_1 - \alpha_1 k_2) + r_1 r_2(r_2 - \alpha_2 k_1) \text{ and}$$
$$c = r_1 r_2(r_1 r_2 - \alpha_1 \alpha_2 k_1 k_2)(r_1 - \alpha_1 k_2)(r_2 - \alpha_2 k_1).$$

Therefore, the equilibrium E_6 is locally asymptotically stable if $R_0^{(2,1)} > 1$ and $R_0^{(2,2)} < 1$.

(viii) The eigenvalues of $\bar{J}(E_7)$ are

$$\lambda_{71} = \left(R_0^{(2,1)} - 1\right)\left(d_1 + \frac{r_1 p N_2^*}{k_1} + \frac{r_1 N_1^*}{k_1}\right),$$
$$\lambda_{72} = b_2\left(1 - R_0^{(2,2)}\right) \text{ and}$$
$$\lambda_{73,74} = -\frac{1}{2}\frac{1}{r_1 r_2 - \alpha_1 \alpha_2 k_1 k_2}\left(b^2 \pm b\sqrt{1 - \frac{4c}{b^2}}\right),$$

where,

$$b = r_1 r_2(r_1 - \alpha_1 k_2) + r_1 r_2(r_2 - \alpha_2 k_1) \text{ and}$$
$$c = r_1 r_2(r_1 r_2 - \alpha_1 \alpha_2 k_1 k_2)(r_1 - \alpha_1 k_2)(r_2 - \alpha_2 k_1).$$

Therefore, the equilibrium E_7 is locally asymptotically stable if $R_0^{(2,1)} < 1$ and $R_0^{(2,2)} > 1$.

(ix) The eigenvalues of $\bar{J}(E^*)$ are

$$\lambda_{*1} = \left(1 - R_0^{(2,1)}\right)\left(d_1 + \frac{r_1 p N_2^*}{k_1} + \frac{r_1 N_1^*}{k_1}\right),$$
$$\lambda_{*2} = b_2\left(1 - R_0^{(2,2)}\right) \text{ and}$$
$$\lambda_{*3,*4} = -\frac{1}{2}\frac{1}{r_1 r_2 - \alpha_1 \alpha_2 k_1 k_2}\left(b^2 \pm b\sqrt{1 - \frac{4c}{b^2}}\right),$$

where,

$$b = r_1 r_2(r_1 - \alpha_1 k_2) + r_1 r_2(r_2 - \alpha_2 k_1) \text{ and}$$
$$c = r_1 r_2(r_1 r_2 - \alpha_1 \alpha_2 k_1 k_2)(r_1 - \alpha_1 k_2)(r_2 - \alpha_2 k_1).$$

Therefore, the equilibrium E^* is locally asymptotically stable if $R_0^{(2,1)} > 1$ and $R_0^{(2,2)} > 1$.

□

Supplementary basic reproductive number

In this section, we are going to calculate the four basic reproductive numbers of our system using the spectral radius of the next-generation matrix. Let us remember that

- $R_0^{(1,1)}$ denotes the basic reproductive number of the predator disease in the absence of the prey.
- $R_0^{(1,2)}$ denotes the basic reproductive number of prey disease in the absence of the predator.
- $R_0^{(2,1)}$ denotes the basic reproductive number of the predator disease in the presence of the prey.
- $R_0^{(2,2)}$ denotes the basic reproductive number of prey disease in the presence of the predator.

Following the notation of van den Driessche and Watmough (2002), it is necessary to decompose the infected compartments of the IGP model with disease as a sum of the infection terms $\mathscr{F}$ and the transition terms $\mathscr{V}$. In the case of the disease in the predator, we denote $\mathscr{F}_1 = \beta_1 S_1 I_1$, and $\mathscr{V}_1 = d_1 I_1 + \frac{r_1}{k_1} I_1(N_1 + pN_2)$, then

$$\frac{dI_1}{dt} = \mathscr{F}_1(S_1, I_1, S_2, I_2) - \mathscr{V}_1(S_1, I_1, S_2, I_2). \tag{1}$$

First we calculate the $R_0^{(1,1)}$. For this, we use the disease-free equilibrium of predator in the absence of prey $E_1(k_1, 0, 0, 0)$ to obtain the following expressions

$$F^{(1,1)} = \frac{\partial \mathscr{F}_1}{\partial I_1}(k_1, 0, 0, 0) \text{ and } V^{(1,1)} = \frac{\partial \mathscr{V}_1}{\partial I_1}(k_1, 0, 0, 0),$$

from which we obtain that $F^{(1,1)} = \beta_1 k_1$ and $V^{(1,1)} = b_1$, then

$$R_0^{(1,1)} = F^{(1,1)} \left(V^{(1,1)} \right)^{-1} = \frac{\beta_1 k_1}{b_1}.$$

Now, we calculate $R_0^{(2,1)}$, in this case the disease-free equilibrium is $E5(N_1^*, 0, N_2^*, 0)$. Using Equation 1, we define $F^{(2,1)}$ and $V^{(2,1)}$ as follows

$$F^{(2,1)} = \frac{\partial \mathscr{F}_1}{\partial I_1}(N_1^*, 0, N_2^*, 0) \text{ and } V^{(2,1)} = \frac{\partial \mathscr{V}_1}{\partial I_1}(N_1^*, 0, N_2^*, 0),$$

then,

$$F^{(2,1)} = \frac{\beta_1 k_1 r_2 (r_1 - \alpha_1 k_2)}{r_1 r_2 - \alpha_1 \alpha_2 k_1 k_2} \text{ and } V^{(2,1)} = d_1 + \frac{r_1 k_1 r_2 (r_1 - \alpha_1 k_2)}{k_1 (r_1 r_2 - \alpha_1 \alpha_2 k_1 k_2)} + \frac{r_1 p k_2 r_1 (r_2 - \alpha_2 k_1)}{k_1 (r_1 r_2 - \alpha_1 \alpha_2 k_1 k_2)}.$$

Rewriting the above expressions we have $F^{(2,1)} = k_1 \beta_1 N_1^*$ and $V^{(2,1)} = d_1 k_1 + r_1 N_1^* + p r_1 N_2^*$. Therefore, the basic reproductive number is

$$R_0^{(2,1)} = F^{(2,1)} \left(V^{(2,1)} \right)^{-1} = \frac{k_1 \beta_1 N_1^*}{d_1 k_1 + r_1 N_1^* + p r_1 N_2^*}.$$

In the case of the disease in the prey, we denote $\mathscr{F}_2 = \beta_2 S_2 I_2$, and $\mathscr{V}_2 = d_2 I_2 + \frac{r_2}{k_2} I_2(N_2 + qN_1) + a I_2 N_1$, then

$$\frac{dI_2}{dt} = \mathscr{F}_2(S_1, I_1, S_2, I_2) - \mathscr{V}_2(S_1, I_1, S_2, I_2). \tag{2}$$

First we calculate the $R_0^{(1,2)}$. For this, we use the disease-free equilibrium of prey in the absence of predators $E_2(0, 0, k_2, 0)$ to obtain the following expressions

$$F^{(1,2)} = \frac{\partial \mathscr{F}_2}{\partial I_2}(0, 0, k_2, 0) \text{ and } V^{(1,2)} = \frac{\partial \mathscr{V}_2}{\partial I_2}(0, 0, k_2, 0),$$

from which we obtain that $F^{(1,2)} = \beta_2 k_2$ and $V^{(1,2)} = b_2$, then

$$R_0^{(1,2)} = F^{(1,2)} \left(V^{(1,2)} \right)^{-1} = \frac{\beta_2 k_2}{b_2}.$$

Now, we calculate $R_0^{(2,2)}$, in this case the disease-free equilibrium is $E5(N_1^*, 0, N_2^*, 0)$. Using Equation 2, we define $F^{(2,2)}$ and $V^{(2,2)}$ as follows

$$F^{(2,2)} = \frac{\partial \mathscr{F}_2}{\partial I_2}(N_1^*, 0, N_2^*, 0) \text{ and } V^{(2,2)} = \frac{\partial \mathscr{V}_2}{\partial I_2}(N_1^*, 0, N_2^*, 0),$$

then,

$$F^{(2,2)} = \frac{\beta_2 k_2 r_1 (r_2 - \alpha_2 k_1)}{r_1 r_2 - \alpha_1 \alpha_2 k_1 k_2} \text{ and } V^{(2,2)} = d_2 + \frac{r_1 r_2 (r_2 - \alpha_2 k_1)}{r_1 r_2 - \alpha_1 \alpha_2 k_1 k_2} + \frac{\alpha_2 k_1 r_2 (r_1 - \alpha_1 k_2)}{r_1 r_2 - \alpha_1 \alpha_2 k_1 k_2}.$$

Therefore, the basic reproductive number is

$$R_0^{(2,2)} = F^{(2,2)} \left(V^{(2,2)} \right)^{-1} = \frac{\beta_2 k_2 r_1 (r_2 - \alpha_2 k_1)}{b_2 (r_1 r_2 - \alpha_1 \alpha_2 k_1 k_2)} = \frac{\beta_2 N_2^*}{b_2}.$$

Supplementary numerical values

The following table shows the numerical values that are used as examples in the simulations.

Table 1. Proposed parameters for the model

	Parameters												
	Initial condition	b_1	d_1	b_2	d_2	p	q	k_1	k_2	ε	a	β_1	β_2
E_1	$(15,10,16,10)$	1.8	0.7	1	0.1	0.5	0.5	70	300	0.2	0.02	0.02	0.001
E_2	$(15,10,16,10)$	1.8	0.7	1	0.1	0.5	0.5	70	300	0.2	0.01	0.02	0.001
E_3	$(40,30,7,5)$	1.8	0.7	1	0.1	0.5	0.5	70	300	0.2	0.03	0.04	0.001
E_4	$(7,5,40,30)$	1.8	0.7	1	0.1	0.5	0.5	70	300	0.2	0.01	0.01	0.01
E_5	$(70,12,45,12)$	1.2	0.2	1.8	0.4	0.5	0.5	70	150	0.2	0.01	0.005	0.002
E_6	$(38,24,30,3)$	1.8	0.5	1.2	0.1	0.5	0.5	70	150	0.2	0.01	0.1	0.001
E_7	$(38,10,38,24)$	1.2	0.2	1.8	0.4	0.5	0.5	70	150	0.2	0.01	0.001	0.09
E^*	$(10,6,30,9)$	1.2	0.2	1.8	0.4	0.5	0.5	70	150	0.2	0.01	0.1	0.1
